# Case Report: Assessing COVID-19 Transmission in Professional Volleyball in Germany, September to December 2020: An Epidemiological Study

**DOI:** 10.3389/fspor.2022.827005

**Published:** 2022-06-14

**Authors:** Oliver Morath, Friedrich Barsch, Adhara Lazaro, Daniela Huzly, Peter Deibert

**Affiliations:** ^1^Department of Medicine, Faculty of Medicine, Institute of Exercise and Occupational Medicine, Medical Center-University of Freiburg, Freiburg, Germany; ^2^Institute of Virology, Freiburg University Medical Center, Faculty of Medicine, University of Freiburg, Freiburg, Germany

**Keywords:** team sport, virus transmission, epidemiology, behavior strategies, SARS-CoV-2

## Abstract

**Introduction:**

The SARS-CoV-2 pandemic poses extraordinary challenges in all fields of daily life. The WHO recommended social distancing guidelines and person-to-person contact was strongly discouraged to contain transmission. Team-based sports were questioned and widely debated. However, there is a lack of available evidence on the risk of in-game SARS-CoV-2 transmission. We aim to derive new insights regarding the risk of SARS-CoV2 infection during team sports and provide current opinion on how to behave during training and competition.

**Methods:**

We report on six competitive male volleyball players (national level) of the same team who were infected with COVID-19. The mode of transmission and potential virus spreading within the team was investigated. The entire course of infection was documented by detailed medical history of the players and RT-PCR tests confirmed suspected infections. Serological testing was performed to establish the antibody status of the team.

**Results:**

The investigation did not show strong evidence of viral transmission within the team during sporting activities. Only two players with PCR-proven infection hat detectable antibodies in two antibody assays.

**Conclusion:**

Private and social gatherings can spread infection into team sports. Clearly defined test strategies and strict adherence to standard COVID-19 hygiene guidelines during sports seasons cannot be overemphasized.

## Introduction

In December 2019 a novel viral infection caused by severe acute respiratory syndrome coronavirus 2 (SARS-CoV-2) was first detected in Wuhan, China (Zhu et al., 2020). The primary mode of transmission is respiratory, mainly via droplets, albeit aerosols have also been implicated to play a role even from asymptomatic persons (Meyerowitz et al., 2020). This led to a rapid worldwide spread of the virus. On March 11, 2020, the WHO declared the situation as a pandemic and a global public health emergency. The range of Coronavirus Disease 2019 (COVID-19) symptoms can vary and manifest as mild flu-like symptoms to severe cases with multiorgan involvement leading to death (Guan et al., 2020; Li and Ma, 2020; Zhu et al., 2020).

The corona pandemic confronted the world of sports with an unprecedented challenge. In particular, team-based sports were questioned in the context of potential virus spreading. Team and contact sports were discouraged due to the high risk of transmission (Steinacker et al., 2020). This led to significant restrictions in the sporting activities among athletes. International sports events were postponed (e.g., Tokyo Olympics, 2020) and major German sports leagues were canceled and deferred. Gyms, sport clubs, and other sport facilities had to close (Wackerhage et al., 2020). Meanwhile, several varying recommendations for sports under pandemic conditions have been developed and most team sports in Western Europe (e.g., German Football Bundesliga May, 2020) have temporarily resumed (Bloch et al., 2020; Carmody et al., 2020). The German Volleyball Bundesliga tournament restarted their season (September 2020) based on specific hygiene guidelines (Konzept Wiederaufnahme Trainings- und Spielbetrieb, 2020). Nevertheless, there is still a lack of systematic data on how team and contact sports contribute to the risk of SARS-CoV-2 infection during training and competition (Hull et al., 2020; Nieß et al., 2020). There are few reports on possible transmission during sports events; however, evidence is still limited (Atrubin et al., 2020; Brlek et al., 2020).

## Methods

This report investigates a total of nine matches of a German male Volleyball Bundesliga (2nd Division) team from September to December 2020 following the first enforced COVID-19 lockdown. We investigated 15 players and staff (i.e., trainer and physiotherapist) who participated up to six times per week in training and matches. The age of the players ranged from 18 to 33 years. Twenty-four hours prior to every match a rapid antigen test (NADAL^®^ COVID-19 Ag plus Test, nal von minden GmbH, Moers, Germany or Clungene Covid-19 Antigen Rapid Test, Hangzhou Clongene Biotech Co., Ltd., Hangzhou, China) was performed according to the guidelines of German Volleyball Bundesliga (Konzept Wiederaufnahme Trainings- und Spielbetrieb, 2020). The rapid antigen test was performed as oropharyngeal swab according to manufacturer information. The course of infection was documented by detailed medical history among other focussing on high-risk contacts and recreational behavior besides training. RT-PCR tests using pooled nasopharyngeal and oropharyngeal swab were administered among suspected athletes. Serological examination was performed with approval of the ethics committee (reference code: 21-1326) and personal written consent of all 15 players and staff.

Additionally, serological tests with three different SARS-CoV-2 antibody assays (i.e., Euroimmun Anti-SARS-CoV-2 ELISA IgG, Euroimmun, Lübeck, Germany; Mikrogen recom Well SARS-CoV-2 IgG, Mikrogen, Martinsried Germany; and Siemens SARS-CoV-2 Total Assay, Siemens Healthcare, Erlangen) measuring antibodies against the S1 domain (Euroimmun), the nucleoprotein (Mikrogen) or the receptor binding domain (Siemens) were performed. All 15 subjects who regularly participated in training sessions were tested. The serological testing took place at least 6 weeks after onset of infection of Player 6.

## Results

In compliance with the club hygiene guidelines, all players wore face masks before and after the training sessions, when entering and leaving the gym, and during physiotherapy treatments. Face masks were not worn only when exercising. Direct contact with other teammates (e.g. gathering during breaks, distance to teammates during warm-up exercises) during training sessions was reduced to a minimum.

On 19th of September 2020, a league game was played in Freiburg, Germany. The first case of SARS-CoV-2 infection likely occurred in a social gathering in a bar after the game, where 8 of the 15 players were present (Day 1). The index patient was unknown. On Day 3, all 15 players went to training and remained asymptomatic. On Day 4, the first player (P1) presented with fever of up to 38.5 °C (oral self-measurement), malaise, and pain in the upper and lower extremities. The following day (Day 5), the second player (P2) developed similar symptoms with fever of up to 38.5°C (oral self-measurement). The third player (P3) reported malaise, fever, and chills on Day 6. At this point, all players and contact persons were quarantined. All three players tested positive using pooled nasopharyngeal and oropharyngeal swabs and RT-PCR method. They (P1-3) were symptom-free on Day 8.

On Day 9, the whole team of 15 subjects was tested with RT-PCR and two additional asymptomatic players were found to be positive (P4 and P5). Both were present at the social gathering and other sources of transmission had been ruled out according to players' amnestic data.

After 14 days of quarantine, the team gradually resumed training. All SARS-CoV-2-positive players had a sports medicine testing in accordance with the recommendation of the German Society of Sports and Science (Nieß et al., 2020) which consisted of medical history, physical examination, laboratory workup, resting ECG, echocardiography, and spiroergometry at our institute. All results were normal. Consequently, the players were released for return-to-sports procedure. All players were instructed to report to the institute in case of symptom recurrence.

**Figure 1:**
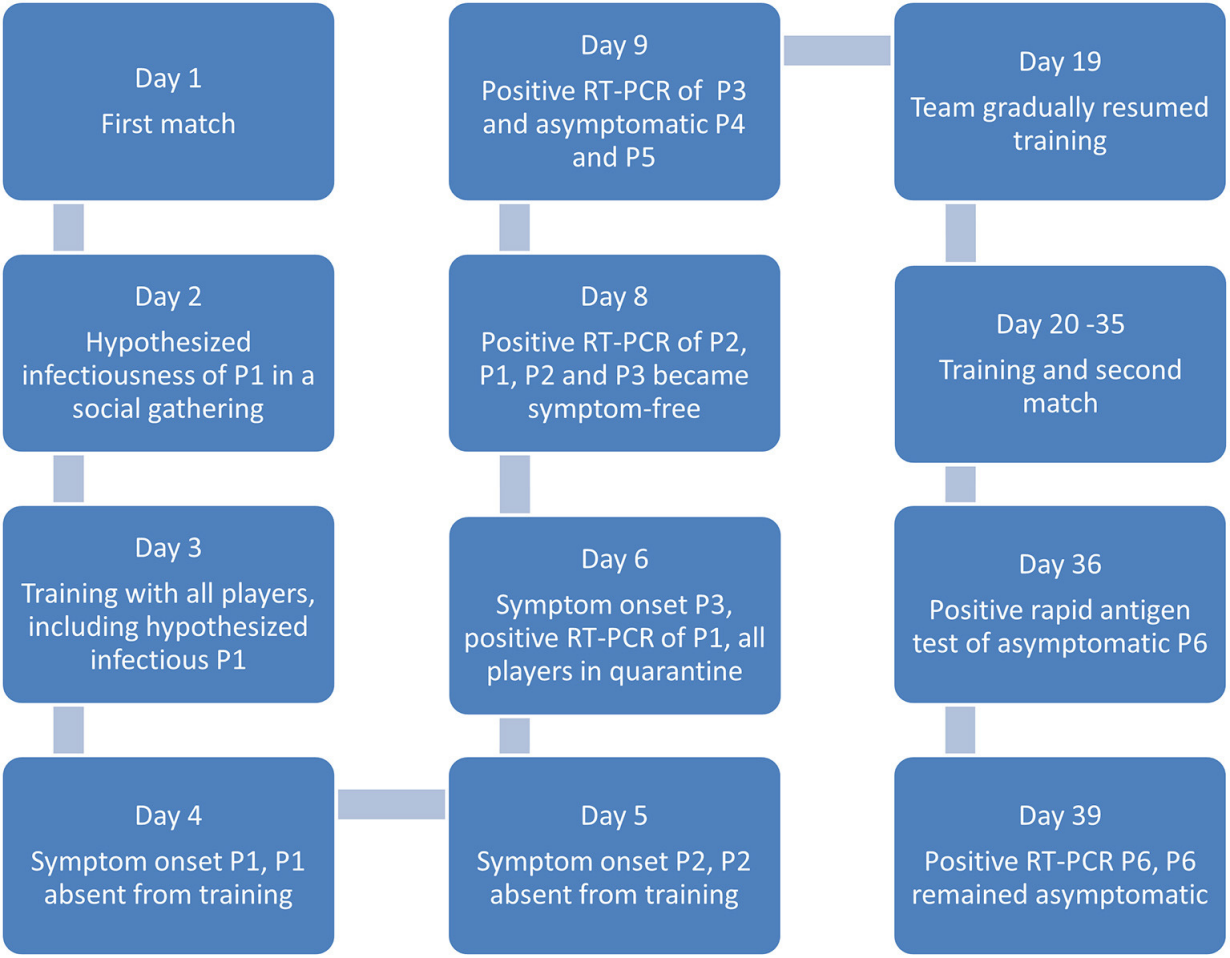
Course of infection within the team.

The volleyball team carried on with training for the succeeding 2 weeks. On Day 36, another asymptomatic player (P6) showed a positive rapid antigen test prior to a league game. This was confirmed positive by RT-PCR on Day 39. In accordance with the local health department guidelines, all players went into quarantine for the second time. No training session was carried out. All players and contact persons (except P1–P5) underwent another RT-PCR test. With the exception of P6, all other tested persons remained negative. The infection of P6 was likely attributed to his contact with a visitor who came from a COVID-19-risk area abroad. The said visitor who complained of episodes of headaches also tested positive on Day 39. P6 remained asymptomatic. Figure 1 shows the course of infection within the team.

Only two players with PCR-proven infection (P2 and P6) had detectable antibodies in two antibody assays. Five of six were positive in the Siemens total antibody assay; two positive and three equivocal results in Mikrogen N antibody assay, and three positives in Euroimmun S1 antibody assay. Results of the antibody immunoassays are shown in Table 1.

**Table 1:** Summary of RT-PCR and serological testing.

**Player**	**RT-PCR**	**Euroimmun S1-IgG**	**Mikrogen**	**Siemens CoV2T** **(RBD total antibody)**	**Final Interpretation**
P1	pos	0.474/neg	20.4/greyzone	2.15/pos	Proven SARS-CoV-2-Infection
P2	pos	3.1/pos	103/reactive	not done	Proven SARS-CoV-2-Infection
P3	pos	0.474/neg	12,9/nonreactive	1.34/pos	Proven SARS-CoV-2-Infection
P4	pos	0.703/neg	22/greyzone	>10.00/pos	Proven SARS-CoV-2-Infection
P5	pos	0.783/neg	17.5/greyzone	2.23/pos	Proven SARS-CoV-2-Infection
P6	pos	1.75/pos	87.7/reactive	not done	Proven SARS-CoV-2-Infection
P7	neg	1.20/pos	8.98/nonreactive	not done	Evidence of past SARS-CoV-2-Infection
P8	neg	0.157/neg	1.94/nonreactive	0.55/neg	No evidence of past infection
P9	neg	0.123/neg	2.74/nonreactive	0.72/neg	No evidence of past infection
P10	neg	0.655/neg	44.9/reactive	1.3/pos	Evidence of past SARS-CoV-2-Infection
P11	neg	0.111/neg	1.94/nonreactive	0.55/neg	No evidence of past infection
P12	neg	0.164/neg	1.45/nonreactive	0.74/neg	No evidence of past infection
P13	neg	0.057/neg	2.58/nonreactive	0.25/neg	No evidence of past infection
P14	neg	0.295/neg	13.5/nonreactive	0.12/neg	No evidence of past infection
P15	neg	0.123/neg	2.15/negative	2.15/pos	No evidence of past infection

Furthermore, P7 and P10 were found to have detectable antibodies in two of three assays. P7 had a known SARS-CoV-2 infection in March 2020 which occurred independently of the outbreak in the team. P10 had neither confirmed infection nor symptoms during the entire season and the past months before the season started. Since no earlier serum sample of P10 was available, the estimated date of infection remained unclear. All other players tested negative in all three antibody assays, ruling out past SARS-CoV-2 infection.

## Discussion

This epidemiological observation reports on six athletes of a national volleyball team infected with SARS-CoV-2. Using supplementary serological testing, one additional previously unknown infection was detected. The viral transmission among the first five players likely occurred in a private gathering after a league game, which 8 of 15 players attended. Although the index patient was not clearly identified by the local health department, it was hypothesized that all infected players contracted the virus from the same index patient. Those players who were not present in the said social gathering were not infected despite having participated in training sessions prior to symptom onset of P1, P2, and P3. However, since P1 was present during training on Day 3, it was not possible to completely rule out that transmission could have occurred during training. All players wore face masks according to club hygiene guidelines, except during exercising when players did not wear face masks. Findings from several published studies suggest that presymptomatic viral transmission 2 days prior to symptom onset is possible (Ganyani et al., 2020; He et al., 2020). Regarding infection of P4 and P5, it was likely that they were infected by the same index patient since all SARS-CoV-2-positive players were present at the private gathering and other sources of transmission had been ruled out on the basis of the athletes' anamnestic data. The infection of P6 was attributed to the SARS-CoV-2-positive visitor who came from a COVID-19-risk area abroad. P7 had a known infection in March 2020. Therefore, the low positive antibody result of P7 could likely be linked to the previous infection in March 2020. Additionally, serological testing showed SARS-CoV2- antibodies in P10, indicating a previous infection. RT-PCR tests of P10 during the outbreak in the team remained negative. The negative RT-PCR tests of P10 could be linked to the protective effect of antibodies developed after the previous SARS-CoV-2 infection in March 2020. Neutralizing antibodies can be found up to 12 months after SARS-CoV-2 infection and prevent from another SARS-CoV-2 infection (Khoury et al., 2021; Wang et al., 2021).

Volleyball is an interval sport with both aerobic and anaerobic components (Künstlinger et al., 1987). High intensity periods can be accompanied by heavy and deep breathing and short team gatherings during volleyball rallies are common. Contacts while playing are short and are mostly seen during block-attack situations and while tactical team gatherings between the rallies. To date, only limited studies exist regarding SARS-CoV-2 transmission associated with specific team sport competitions and practices. Atrubin et al. documented a SARS-2-CoV transmission during a recreational ice hockey game in Florida (Atrubin et al., 2020). It was reported that one symptomatic player infected twelve other participants during an indoor sporting activity. The index patient showed symptoms the day after the game suggesting that the disease's early phase is the period of highest infectiousness (He et al., 2020; To et al., 2020). Furthermore, since it has been shown that corona viruses survive significantly longer in cold air and at lower humidity, the risk of infection can be increased under these conditions (Ijaz et al., 1985; Aboubakr et al., 2020). An emission rate study by Buonanno et al. showed that asymptomatic SARS-CoV-2 patients could cause high viral emissions by oral breathing during light and heavy exercises increasing the risk of airborne transmission (Buonanno et al., 2020). This finding suggested a possible higher risk of viral transmission in contact sports.

Serological testing can be a useful diagnostic tool to supplement PCR tests among suspected individuals since antibody response after SARS-CoV-2 infection is common (Deeks et al., 2020; Long et al., 2020; Zhao et al., 2020). Nevertheless, detectable antibody levels have shown to decline in several individuals and seroreversion is not uncommonly seen (Self et al., 2020). IgG antibody testing instead of IgM or IgA testing were used due to better accuracy as recommended by the Infetious Diseases Society of America (Hanson et al., 2022). Additionally, antibody assays have varying sensitivities and specificities (Deeks et al., 2020). Using different assays targeting antibodies against different epitopes of the virus can help overcome this problem. Three different antibody assays were used, however even the assay with the highest sensitivity (i.e., Siemens SARS CoV-2 total assay) showed nonreactive result in one of seven players with PCR-proven infection. Thus, it is difficult to draw conclusions on the infection status of individuals with negative antibody titers. Furthermore, it was not possible to determine the index patient of P10 by using serological testing alone. Therefore, a transmission during the team outbreak, despite negative RT-PCR tests, could not be completely ruled out. Regarding the decline of the antibody levels and the low antibody levels of P10, it can be assumed that the infection might have previously occurred (Self et al., 2020).

Serological testing might be a helpful tool for diagnosing previous SARS-CoV-2 infections, however there are some limitations. Test selection is important since different antibody test kits yield different results and demonstrate varied sensitivities and specificities. Moreover, timing of testing and positive predictive value should be considered (Deeks et al., 2020; CDC, 2021). This finding is consistent with the current recommendations of the guidelines for COVID-19 antibody testing of the CDC (CDC, 2021).

Another study investigated the risk of viral transmission in British Super League rugby matches with SARS-CoV-2 positive players. Jones et al. did not demonstrate any in-game transmission and reported that SARS-CoV-2 infections were attributable to transmissions outside of the sporting activity indicating that the risk of viral transmission during outdoor sporting activities might not be increased (Jones et al., 2021). Dixon et al. investigated the risk of viral transmission in US college football games. They showed among 1190 athletes and a total of 64 games no infection of other players, despite having contact with a positive player (Dixon et al., 2021). Two other studies used video recordings of soccer matches to investigate the contact times of players. The risk of transmission was found to be low taking into account that soccer is an outdoor sport (Egger et al., 2021; Faude et al., 2022).

In contrast to the findings on outdoor sports, indoor activities might be on higher risk for viral transmission. Pauser et al. report on with 36 infected persons after a basketball match. Routine testing strategies were not applied. They conclude that risk of viral transmission in indoor sports is higher compared to outdoors and that adherence to hygiene guidelines (i.e., wearing facemasks) if of high importance (Pauser et al., 2021).

A recently published study by Mack et al. reported on prospective testing during the resumption of the 19/20 NBA season in the “2020 NBA Bubble” in Orlando. Testing resulted in a total of 36 persistent positive cases. All persistent positive tested individuals were asymptomatic and case monitoring including PCR testing was continued for up to 100 days. The authors of this study stated that no new infections occurred after the quarantine ended in Orlando, indicating effective hygiene guidelines (Mack et al., 2021). A case report from South Korea described an outbreak in fitness dance classes where 57 patients were infected, suggesting that high-intensity activities might increase the risk of viral transmission (Jang et al., 2020). In Slovenia, a cluster of SARS-CoV-2 infections of five squash players was reported wherein a possible indirect transmission via contaminated objects was suggested. The exact mode of transmission in this case remained unclear (Brlek et al., 2020). Both case reports were consistent with the findings that indoor activities are major risk factor for viral transmission (Qian et al., 2020).

Another investigation of high-level male soccer professionals showed IgG-Antibodies in 18 out of 30 athletes (Gervasi et al., 2020). Six of the 18 were asymptomatic at all times. In that study, it was not discussed how transmission and infection of the athletes might have occurred; however, the six asymptomatic athletes with positive IgG-Antibodies might suggest a risk of viral transmission during training and competition.

Limitations of this study include the inability to identify the index patient. Consequently, it was not possible to perform SARS-CoV-2 genome sequencing to fully track the course of infection and draw conclusions on how the transmission occurred. This could have provided more evidence whether the risk of viral transmission during sporting activity might be higher compared to private gatherings. In addition, this study is limited by the small sample in a relatively short time frame. A prospective study design with consequent infection monitoring via RT-PCR could provide more specific data to confirm player-to-player transmission. Furthermore, it has to be acknowledged that all conclusions drawn are limited to the small sample size of that case. By having a bigger sample size observed over a longer time period, it would be possible to draw more evident conclusions.

Additionally, players should also be aware of the typical clinical picture of Covid-19 disease and carefully monitor themselves in this regard. If symptoms occur, they should immediately inform their team managers in a timely manner and prompt testing should be initiated. Until negative test results are obtained, the suspected players should remain in quarantine. Moreover, regular testing should be initiated. In Deutsche Fußball-Bundesliga, depending on the national incidence level, two PCR tests per week are required. The authors support and consider these said guidelines reasonable for team sports and competitions. If PCR testing is not feasible, we recommend regular rapid antigen testing on a daily base, but at least twice a week. Rapid antigen test is easy to perform and has shown high acceptance in athletes (Finch et al., 2022). Recommending a more frequent testing strategy goes in line with the findings of Kamo et al., who investigated the effectiveness of different COVID-19 testing strategies. They stated that frequently up to daily testing by PCR can reduce the number of SARS-CoV-2 infected athletes by almost 80 % and that daily testing is an effective measure strategy (Kamo et al., 2022). Mina et al. underlined the importance of high frequently testing (Mina et al., 2020). Still, further research in transmission risk during training, competition, and other contact sport settings are needed to further elucidate the findings presented. Therefore, prospective studies investigating the risk of transmission by tracking contact times (i.e., wearing tracking devices) combined with serial PCR testing could help elucidate the risk of viral transmission in team sports.

## Conclusion

This investigation provides evidence that SARS-CoV-2 transmission during private and social gatherings can spread infection into team sports. A conclusive statement on how sporting activity affects viral transmission cannot be drawn based on this study. Professional athletes should strictly adhere to the recommended hygiene guidelines and regulations and avoid non-essential interpersonal contacts in order to prevent the spread of infection within the team and training partners. It is recommended that coaches and support staff should strongly advice the players against participating in private gatherings in order not to unnecessarily compromise the training sessions and competitions.

In conclusion, our findings show that SARS-CoV-2 transmission during private and social gatherings can spread infection into team sports underlining the importance of test strategies during sports season and strict adherence to COVID-19 hygiene guidelines.

## Data Availability Statement

The original contributions presented in the study are included in the article/Supplementary Material, further inquiries can be directed to the corresponding authors.

## Ethics Statement

The studies involving human participants were reviewed and approved by Ethics Committe of Albert-Ludwigs-University of Freiburg (reference code: 21-1326). The patients/participants provided their written informed consent to participate in this study.

## Author Contributions

OM, FB, and PD contributed to the conception and design of the study. OM performed the blood collections and rapid antigen test. FB performed the PCR specimen collection. DH performed all virological testings. OM wrote the first draft of the manuscript. AL wrote sections of the manuscript. All authors contributed to manuscript revision, read, and approved the submitted version.

## Conflict of Interest

The authors declare that the research was conducted in the absence of any commercial or financial relationships that could be construed as a potential conflict of interest.

## Publisher's Note

All claims expressed in this article are solely those of the authors and do not necessarily represent those of their affiliated organizations, or those of the publisher, the editors and the reviewers. Any product that may be evaluated in this article, or claim that may be made by its manufacturer, is not guaranteed or endorsed by the publisher.
